# Therapeutic Strategy in Low-Risk Papillary Thyroid Carcinoma – Long-Term Results of the First Single-Center Prospective Non-Randomized Trial Between 2011 and 2015

**DOI:** 10.3389/fendo.2021.718833

**Published:** 2021-09-06

**Authors:** Agnieszka Czarniecka, Marcin Zeman, Grzegorz Wozniak, Adam Maciejewski, Ewa Stobiecka, Ewa Chmielik, Malgorzata Oczko-Wojciechowska, Jolanta Krajewska, Daria Handkiewicz-Junak, Barbara Jarzab

**Affiliations:** ^1^The Oncologic and Reconstructive Surgery Clinic, M. Sklodowska-Curie National Research, Institute of Oncology Gliwice Branch, Gliwice, Poland; ^2^Tumor Pathology Department, M. Sklodowska-Curie National Research Institute of Oncology Gliwice Branch, Gliwice, Poland; ^3^Genetic and Molecular Diagnostics of Cancer Department, M. Sklodowska-Curie National Research Institute of Oncology Gliwice Branch, Gliwice, Poland; ^4^Department of Nuclear Medicine and Endocrine Oncology, M. Sklodowska-Curie National Research Institute of Oncology Gliwice Branch, Gliwice, Poland

**Keywords:** low-risk papillary thyroid carcinoma, extent of surgery, prospective trial, risk of relapse, postoperative complications

## Abstract

Optimal therapeutic strategy in low advanced papillary thyroid carcinoma (PTC) is still a matter of debate. The management differs depending on the country.

A prospective non-randomized study was performed to evaluate whether less extensive surgery could be a safe, acceptable, and sufficient therapeutic option in PTC cT1N0M0 patients. The present paper summarizes the results of over a 5-year follow-up

**Material:**

Our prospective group (PG) treated between 2011 and 2015 consisted of 139 patients with cT1aN0M0 PTC who underwent lobectomy (LT) as initial surgical treatment (PGcT1aN0M0 group) and 102 cT1bN0M0 patients in whom total thyroidectomy (TT) with unilateral central neck dissection (CND) was performed (PGcT1bN0M0). PG was compared with the retrospective group (RG) of patients who underwent TT with bilateral CND between 2004 and 2006: 103 cT1aN0M0 patients (RGcT1aN0M0) and 91cT1bN0M0 (RGcT1bN0M0). The risks of reoperation, cancer relapse and postoperative complications were analyzed.

**Results:**

Only 12 cT1aN0M0 patients (7.6%) withdrew from the trial and underwent TT with bilateral CND. Over 90% of patients accepted less extensive surgery. In 4 cT1aN0M0 cases, TT with CND was performed due to lymph node metastases found intraoperatively. The initial clinical stage according to the TNM/AJCC 7^th^ edition was confirmed histologically in 77% of cases in PGT1aN0M0 and in 72% in PGT1bN0M0, respectively. 24 PGcT1aN0M0 patients were reoperated on. In this group, cancer lesions in the postoperative histological specimens were found in 8 cases (32%). Five-year disease-free survival (DFS) was excellent. However, no statistically significant differences were found between PG and RG groups (99.3% in PGcT1aN0M0 and 99.0%, in RGcT1aN0M0; p = 0.41 and 98%, in PGcT1bN0M0 and 94.4% in RGcT1bN0M0; p=0.19). No significant differences were observed in the incidence of early paresis of the recurrent laryngeal nerves between PG and RG. However, as predicted, LT completely eliminated the risk of postoperative hypoparathyroidism.

**Summary:**

The results of the prospective clinical trial confirm that less extensive surgery in adequately selected low-advanced PTC patients is both safe and sufficient.

## Introduction

Currently, papillary thyroid carcinoma (PTC) is a common endocrine neoplasm, and the population of newly diagnosed, low-stage cancers is constantly growing (1). Therefore, current recommendations on the therapeutic strategy (especially in this stage) should be updated and modified to avoid overdiagnosis and overtreatment and to prevent the worsening of treatment results (2). Of note, reduction in adverse effects is also a key aspect of therapy to improve patients’ quality of life (3).

The lack of prospective randomized clinical trials results in the fact that the majority of the current recommendations are based on retrospective studies, meta-analyses, or expert opinions (4). Therefore, for many years, the optimal extent of surgical treatment in differentiated thyroid carcinomas has been a subject of debate (5, 6).

Before 2009, two different groups of recommendations were used in the USA. The guidelines of the National Comprehensive Cancer Network (NCCN) and the Society of Surgical Oncology (SSO) recommended the reduction in the extent of surgery in low-risk groups of patients. In turn, the recommendations of the American Thyroid Association (ATA) and the American Association of Clinical Endocrinologists (AACE), which were similar to the European Thyroid Association (ETA) guidelines and the Polish ones, recommended TT, or near-TT with prophylactic CND in all cases of thyroid cancer diagnosed pre- or intra-operatively.

In 2009, ATA revised the guidelines and recommended only LT in low-risk PTC patients with unifocal PTC of up to 1 cm (T1a) in diameter without the suspicion of lymph node metastases. According to the ATA, prophylactic (ipsi- or bilateral) CND was not necessary in patients staged T1-T2N0M0 (7).

At that time, Polish guidelines for diagnosis and treatment of thyroid cancer recommended TT with CND in all cases of thyroid cancer diagnosed preoperatively (using Fine Needle Aspiration Biopsy; FNA) or intraoperatively (by frozen sections) (8). That was the main reason why in 2011 we started a prospective non-randomized trial in low-advanced PTC patients to evaluate whether less extensive surgery could be a safe and well accepted procedure in PTC cT1N0M0 patients.

Since the end of the trial, much has changed as regards international recommendations for the therapeutic management of low-risk PTC. Since 2015, ATA has recommended LT (also in the case of cT2N0M0) (2). The possibility of active surveillance (AS) is gaining more interest in microcarcinoma (9–12). Additionally, there has been the possibility of using image-guide thermal ablation techniques such as laser ablation (LA), radiofrequency ablation (RFA), microwave ablation (MWA) and high-intensity focused ultrasound (HIFU) not only in patients with benign tumors but also with malignant lesions, including papillary microcarcinoma (13–16).

As a result, it will be necessary to change and update the recommendations on diagnosis and management of PTC in the future. This change is scheduled for 2022 in Poland. Based on the above trial, the current paper summarizes the long-term results of less extensive surgery in cT1N0M0 patients, which resulted in the de-escalation of surgical treatment in our country.

AIM: Assessment of long-term results of the prospective trial in cT1N0M0 PTC patients to evaluate the safety, acceptance, and effectiveness of less extensive surgery.

## Material And Methods

We recruited patients for a prospective nonrandomized clinical trial after obtaining the consent from the Bioethics Committee of the Center of Oncology – The Maria Sklodowska-Curie Memorial Institute (no KB/498-18/11), between 2011 and 2015. The end of the recruitment (in the middle of 2015) was planned in connection with the revision of the Recommendations of the Polish Group for Endocrine Tumors regarding the diagnosis and treatment of patients with thyroid cancer, scheduled for the end of 2015.

During the study, TNM/AJCC 7^th^ edition was used (17). The inclusion and exclusion criteria are given in **Table 1**.

**Table 1:** The inclusion and exclusion criteria from our prospective study.

Inclusion criteria	Exclusion criteria
- age ≥ 18 years - presence of a unifocal change in preoperative ultrasound examination: up to 1 cm for the cT1aN0M0 group or > 1 to 2 cm for the cT1bN0M0 group - papillary thyroid cancer (Bethesda VI) diagnosed by preoperative FNA - diagnosis confirmed by two independent pathologists - lack of clinically or ultrasound suspicious cervical lymph nodes - no other risk factors (e.g. radiotherapy of the neck area) - exclusion of distant metastases - informed written consent to participate (confirmed by a signature on a separate form)	- age < 18 years of age - multifocal changes on ultrasound - tumor diameter > 2 cm on ultrasound - clinical or ultrasound presence of cervical lymph nodes suspected of metastases - pregnancy and lactation - past radiotherapy of the neck area - confirmed distant metastases - lack of consent to participate in the study; the patient could withdraw consent to participate until the day of surgery

In our Institute, enrollment for treatment is always conducted based on the standard protocol. Therefore, each patient underwent neck ultrasound with the lymphatic system assessment and fine needle aspiration biopsy (FNAB). When biopsy was collected outside of our Institute, the specimen was verified by another experienced pathologist. According to the Polish recommendations, only patients whose PTC was diagnosed by FNAB and confirmed by two independent pathologists were referred for surgery. Chest X-ray, evaluation of thyroid function (based on TSH, fT3, fT4), preoperative ENT examination with the assessment of vocal fold mobility and anesthesiological consultation were performed.

Depending on the preoperative assessment, patients were divided into 2 groups (i.e. cT1aN0M0 and cT1bN0M0) that were different in terms of the extent of surgery.

### The Extent of Surgical Treatment

#### cT1aN0M0 Group

Only LT was recommended for patients with unifocal PTC of up to 1 cm in diameter without nodal and distant metastases. Patients were informed about the risk of reoperation or the extent of surgery when a higher stage of the disease was found. It could include TT with CND and diagnostic open surgical lymph node biopsy of the lateral cervical neck compartment (level III) on the side of the cancer with histological assessment (frozen sections), which is a standard procedure in our Institute. In the case of lymph node metastases, modified lateral neck dissection could be possible.

#### cT1bN0M0 Group

In patients with unifocal PTC with the lesion size of > 1 cm <= 2 cm without the involvement of the cervical lymph nodes and distant metastases, the limitation of the extent of surgery consisted in prophylactic unilateral CND (on the tumor side). TT was performed in all cases.

Patients were informed about the possibility of more extensive surgery (i.e., bilateral central neck dissection and uni- or bilateral modified lateral cervical lymphadenectomy) during the first intervention or about the possibility of reoperation.

The first surgery was performed on 13^th^ March 2011, whereas the last surgical procedure on 21^st^ July 2015. Participation in the study was proposed to consecutive 265 patients in whom a preoperative FNA diagnosis revealed PTC (Bethesda VI) (240 women - 90.6% and 25 men - 9.4% aged 18-87 years).

One hundred and sixty one patients were classified to clinical stage of cT1aN0M0 (60.8%) and 104 patients to cT1bN0M0 (39.2%). Postoperative histological diagnosis confirmed PTC in 257 patients (97.0%). Eight patients (3%) were diagnosed with a benign lesion and these patients were excluded from further analysis.

In addition, 12 patients (7.5%) initially qualified to the cT1aN0M0 group refused to participate in the study. In these patients, we performed TT with bilateral prophylactic CND and surgical open biopsy of cervical lateral lymph nodes (compartment III) on the side of the cancer was performed.

Additionally, in 4 (2.5%) patients from the cT1aN0M0 group, the presence of lymph node metastases to the central compartment was confirmed intraoperatively and the extent of surgery was widened. The descriptive analysis was performed for these patients.

Only patients in whom PTC was confirmed postoperatively and who were treated in accordance with the established trial were included in a more detailed analysis.

**Figure 1:**
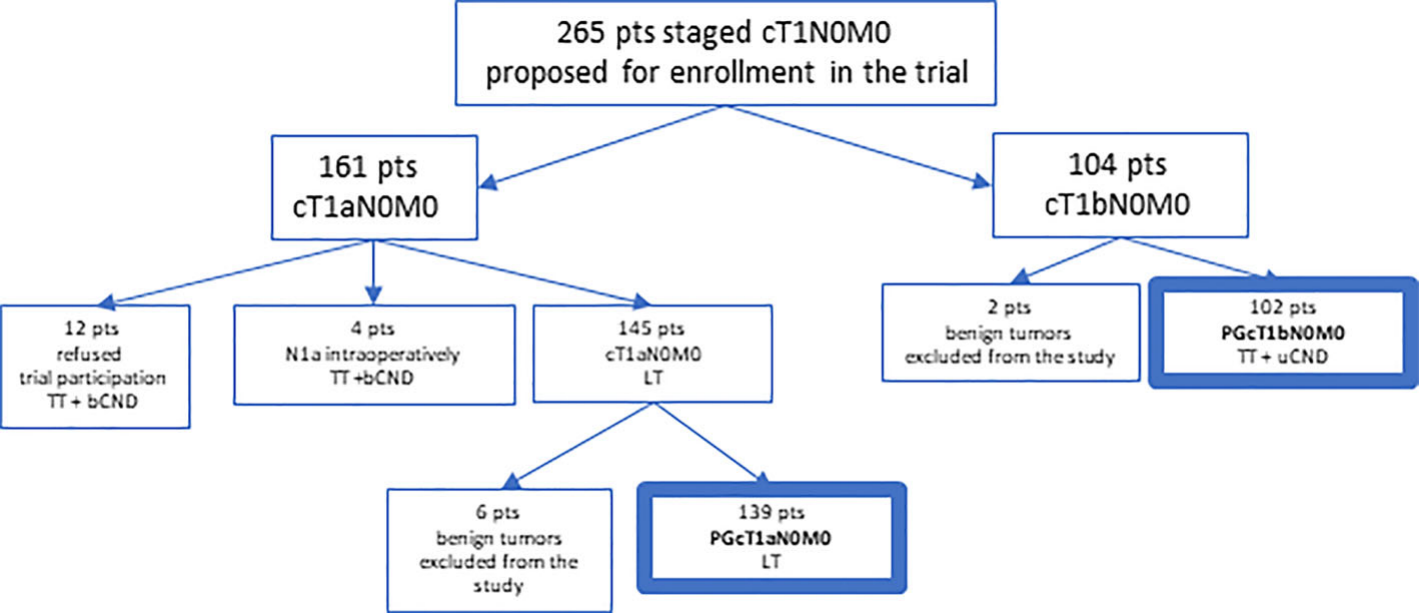
Patient selection to the PG group.

This group was termed prospective (PG) and comprised two subgroups. The first subgroup consisted of 139 patients undergoing LT as initial treatment (PGcT1aN0M0 group) and included 127 women (91.4%) and 12 men (8.6%) (mean age: 39.59, median: 37.22 [18.28-82.81], mean follow-up: 6.72 years, median: 7.1 [1 mo-10.1 years]). The second group included 102 cT1bN0M0 patients (PGcT1bN0M0 group) (92 women [90.2%], 10 men [9.8%], mean age: 45.23, median: 42.67 [21.37-86.6], mean follow-up: 6.69 years, median: 7.1 [1 mo-9.97 years]). Patient selection to the PG group is given in **Figure 1**.

Additionally, to compare the risk of complications and the incidence of relapses at the last stage of the analysis, patients from the PG group were compared with subjects with a similar clinical stage who were retrospectively analyzed, i.e., a retrospective group (RG). Those patients were operated on between 2004 and 2006. All patients underwent TT with bilateral CND. We analyzed and further described the group of 233 consecutive PTC patients in 2015 in terms of the significance of the BRAF mutation for the risk of relapse (18). For the purpose of the current study, from the above group, we selected 103 patients in whom preoperative clinical stage was assessed as cT1aN0M0 (RG cT1aN0M0) and 91 subjects staged cT1bN0M0 (RG cT1bN0M0) whose data were reanalyzed in 2021.

The RGcT1aN0M0 group consisted of 92 women (89.3%) and 11 men (10.7%) (mean age: 45.25 years, median: 43.47 years [18.44 -74.61], mean follow-up: 12.78; median: 13.88 years [2.14-16.84 years]).

The RGcT1bN0M0 group included 92 women (89.0%) and 10 men (11.0%) (mean age: 46.05 years, median: 46.00 years [19.13-92.84], mean: 12.59; median: 14.31 years [3mo-16.83 years]).

In these groups, the incidence of relapses and the assessment of the risk of early postoperative complications were assessed.

Statistical analysis was performed using IBM SPSS Statistics 22 software. Continuous variables were analyzed with the non-parametric Mann-Whitney U test, whereas associations of categorical variables were assessed by the chi-square statistics with the exact Fisher test. The analysis of survival data was performed using the Kaplan-Meier method, with log-rank test comparison between subgroups. The curves were prepared using Statistica 13.1. Statistical significance was adopted at p<0.05.

## Results

The analysis of the results was conducted in several stages. Four different descriptive analyses show the results, depending on the population assessed.

### Stage 1

#### Analysis of cT1aN0M0 Patients Who Underwent TT With Bilateral CND as Primary Treatment

Twelve patients (7.5%) staged cT1aN0M0 refused to participate in the clinical trial. In this group, postoperative advancement (pT1aN0M0) was confirmed in 8 patients (66.7%).

In 2 patients (16.7%), a multifocal cancer was detected (pT1amN0M0).

A single metastasis to the central lymph node was found in 1 patient (tumor diameter of 6mm) (pT1aN1aM0). In 1 patient, infiltration of the thyroid capsule was found, and cancer stage (pT3N0M0) was then diagnosed. (Based on the classification of the TNM/AJCC 8^th^ edition (19), this patient was reclassified as pT1aN0M0 after pathological confirmation).

Eight patients staged pT1aN0M0 were not treated with adjuvant radioactive iodine. The remaining 8 patients received adjuvant radioiodine therapy. In other 4 patients staged cT1aN0M0 (1.9%), TT with CND was performed due to the intraoperative presence of metastases to the central lymph node compartment (confirmed by intraoperative lymph node biopsy). Postoperative histological assessment revealed the following stages of the disease: pT1aN1aM0, pT1amN1aM0, and 2 pT1bN1aM0. Adjuvant treatment with radioactive iodine was given to patients postoperatively.

In a 35-year-old female patient staged pT1bN1aM0 (15 mm in diameter), the disease recurrence was confirmed as lateral cervical lymph node metastasis. She was operated on 30^th^ January 2013 (TT with CND). The postoperative stage of the patient was pT1bN1aM0 and she was given adjuvant 131-iodine therapy (100 mCi). Seven months after the first operation, the patient underwent another surgery due to metastasis to the cervical lymph system, which was confirmed by FNA. On 19^th^ July 2013, modified right-sided cervical lymphadenectomy of II-V levels was performed. The patient was given another 131-iodine therapy. No relapse was observed in a 7.4-year follow-up (6.5-year follow-up after nodal recurrence).

In this group of 16 patients staged cT1aN0M0 who underwent TT with bilateral CND, early postoperative complications included unilateral transient paresis of the vocal fold in 1 patient (6.3%), and hypoparathyroidism (immediately after surgery) in 3 patients (18.8%).

At the first follow-up, one month after surgery, postoperative hypoparathyroidism was observed in 2 patients (12.5%). Of note, no permanent complications were observed.

### Stage 2

#### Analysis of cT1aN0M0 Patients Who Underwent LT as Initial Surgery

LT was performed in 145 patients. Postoperative histological examination showed a proliferative nodule with accompanying thyroiditis in 6 patients (4.1%) who were excluded from further analysis.

The detailed analysis included 139 patients (PGcT1aN0M0) with postoperative cancer diagnosis (PTC - classic variant 109 (78.4%); PTC - follicular variant 28 (20.1%); other: 2 (1.4%) - tall-cell variant, Warthin-like).

Postoperative histological examination revealed pT1a cancer in 120 cases (86.3%).

According to the TNM/AJCC 7^th^ edition, postoperative confirmation of clinical advancement was observed in 107 patients (77.0%) - pT1aN0M0. All patients were staged M0. Detailed characteristics of the PGcT1aN0M0 group is given in **Table 2**.

**Table 2:** Postoperative advancement of pTNM, including multifocal disease (m) in 139 PGcT1aN0M0 patients according to the TNM/AJCC 7^th^ or 8^th^ edition.

TNM/AJCC 7^th^ edition						
pN	pT					
	pT1a	pT1a m	pT1b	pT1b m	pT3	pT3 m
pN0	107 77.0%	9 6.5%	6 4.3%	1 0.7%	8 5.8%	3 2.1%
pN1a	4 2.9%	–	–	–	1 0.7%	–
TNM/AJCC 8^th^ edition						
pN0	115 82.7%	12 8.7%	6 4.3%	1 0.7%	–	–
pN1a	5 3.6%	–	–	–	–	–

Postoperative histology showed central lymph nodes, which were fortuitously found in 53 patients (38.1%) after LT. Metastases occurred in 5 cases (5/53; 9.4%) (**Table 2**).

In 1 patient, a single micrometastasis was detected. However, the patient refused reoperation and was only followed up without cancer relapse. Four patients underwent reoperation (**Table 3**). After LT, multifocal tumor growth in the removed thyroid lobe was observed in 13 patients (9.4%) staged 9 pT1a, 1 pT1b and 3 pT3. Nine patients were reoperated on (**Table 3**).

**Table 3:** Results of reoperation due to a higher stage of the disease after LT in 24 patients from the PGcT1aN0M0 group.

Result of re-operation Cancer presence	Post-LT advancement of the disease according to the TNM/AJCC 7^th^ edition as the cause of reoperation						
	pT1aN1a n=3	pT1amN0 n=6	pT1bN0 n=5	pT1bmN0 n=1	pT3N0 n=6	pT3mN0 n=2	pT3N1a n=1
neo (+)	1	2	1	1	2	1	–
neo (–)	2	4	4	–	4	1	1
	**Post-LT advancement of the disease according to the TNM/AJCC 8^th^ edition as the cause of reoperation**						
	**pT1aN1a** **n=4**	**pT1amN0** **n=6**	**pT1bN0** **n=5**	**pT1bmN0** **n=1**	**pTaN0** **n=6**	–	–
neo (+)	1	3	1	1	2	–	–
neo (–)	3	5	4	–	4	–	–

neo (+) – cancer cells detected in the postoperative assessment.

neo (–) – cancer cells not detected in the postoperative assessment.

##### Summary of the Subgroup of Patients Who Underwent Reoperation

During the trial, TNM/AJCC 7^th^ edition (17) was used, which resulted in reoperation in 24 patients due to the difference in clinical and postoperative disease stage.

In the reoperated group, postoperative histological examination revealed cancer (neo +) in 8 (33%) patients. Detailed characteristics of these patients are presented in **Table 3**.

All patients who underwent completion surgery received adjuvant 131-iodine therapy. Nodal relapse was observed in one subject who was reoperated on due to multifocality with neo (–) findings after another surgery (**Table 4**).

**Table 4:** Detailed description of relapses in the PG group.

feature	PGcT1aN0M0 n=139	PGcT1bN0M0 n=102	
gender, age 1st surgery type: data: postoperative advancement (TNM/AJCC 7^th^ ed.) reoperation relapse date of recurrence surgery final stage 131-iodine treatment (number of therapies, total dose) time to relapse time without relapse	female, aged 29 LT 11^th^ Jan 2012 pT1amN0M0 multifocal cancer yes metastasis to the central lymph nodes 3^rd^ Oct 2012 pT1amN1aM0 yes 2x, 200 mCi 6 mo 7.27 years	female, aged 57.4 TT+uCND 21^st^ Oct 2011 pT1bN1bM0 metastases to the central lymph nodes (4 positive/ 5 removed) no metastasis to the lateral right lymph nodes 16^th^ Feb 2012 pT1bN1bM0 yes 2x, 200 mCi 3 mo 9.49 years	female, aged 29.8 TT+uCND 1^st^ Sept 2014 pT3N0M0 infiltration of the thyroid capsule, negative biopsy of lateral lymph nodes no metastasis to the lateral left cervical lymph nodes 10^th^ Jun 2015 pT3N1bM0 yes 2x, 200 mCi 7 mo 5.06 years

LT-lobectomy; TT+uCND – total thyroidectomy with unilateral central neck dissection.

Postoperative complications were as follows: None of the patients presented with complications after the first operation. After reoperation, one patient presented with transient vocal cord paresis (4%) and early postoperative hypoparathyroidism (PTH <3 pg/ml) was found in 5 patients (20.8%).

After a month, supplementation of calcium and vitamin D was maintained in 2 patients (8.3%).

##### Summary of the Subgroup of Patients Who Refused Reoperation

Another subgroup included patients (n=8) who refused to be reoperated on after LT. Postoperative advancement was as follows: 3 patients with multifocal cancer (pT1amN0M0); 1 patient with the presence of nodal micrometastasis in whom the central node was removed fortuitously (pT1aN1aM0); 1 patient staged pT1bN0M0 and 3 patients staged pT3 (2 pT3N0M0, 1 pT3mN0M0).

According to the TNM/AJCC 8^th^ edition, postoperative staging after pathological reanalysis was as follows: 3 microcarcinoma patients (pT1aN0M0), 4 patients with multifocality (pT1amN0M0), 1 patient with metastases in the central lymph node compartment (pT1aN1aM0), and 1 patient with cancer larger than 1 cm in diameter (pT1bN0M0).

These patients were not treated with adjuvant radioiodine. None of the patients had a recurrence in the mean follow-up (8.65 years); (median 8.91) (range: 5.81-10.1 years). None of the patients presented with early postoperative complications.

In 139 patients (PGcT1aN0M0 group) who underwent LT as the initial treatment, only 1 relapse was observed (**Table 4**). Early paresis of the recurrent laryngeal nerve assessed on day 1 or day 2 postoperatively was noted in 2 patients (1.4%).None of the patients presented with postoperative hypoparathyroidism. Permanent complications were not observed.

### Stage 3

#### Analysis of cT1bN0M0 Patients Treated With TT and Unilateral CND

This group initially consisted of 104 patients. The diagnosis of cancer was not confirmed in 2 patients in the postoperative histological examination (1.85%).

A detailed analysis was conducted in 102 patients with postoperative PTC diagnosis (PGcT1bN0M0 group).

The classic type of PTC was prevalent and diagnosed in 79 patients (77.5%). In 21 subjects (20.6%), the follicular variant was diagnosed. Other variants (diffuse sclerosing and solid) were confirmed in 2 patients (2.0%).

Clinical advancement was confirmed in 72.1% of patients (71 pT1b patients). In 5 patients (4.8%) cancer was diagnosed at a lower stage (pT1a), in 2 patients as pT2 (2%) and infiltration of the thyroid capsule was reported in 24 patients (23.1%; pT3). Eighty five patients (81.7%) received adjuvant therapy with radioactive iodine. Multifocality was observed in 24 subjects (23.5%) and the related characteristics are given in **Table 5**.

**Table 5:** Comparison of postoperative advancement (TNM/AJCC 7^th^ edition) of the disease in the PG group.

	PGcT1aN0M0 n=139	PGcT1bN0M0 n=102
Histological variants of PTC: classic variant follicular variant other	109 (78.4%) 28 (20.1%) 2 (1.5%)	79 (77.5%) 21 (20.5%) 2 (2.0%)
Multifocality - depending on pT (AJCC TNM 7^th^ edition)	13 (9.4%) 9 pT1a 1 pT1b 3 pT3	24 (23.5%) 1 pT1a 14 pT1b 9 pT3
	p=0.036	
N feature	5/139 (3.6%) Lymph nodes were assessed in 53 of 139 patients (38%)	24 (23.1%)
Metastases to the central lymph node compartment	5 (5/53) (9.4%)	18 (17.3%)
Metastases to the lateral cervical lymph node compartment	0	6 (5.7%)
	p=0.033 p=0.000	
M feature	0	0

In PGcT1bN0M0, lymph node metastases were found in 24 patients (23.5%), i.e., 19 patients with pT1b and 5 patients with pT3. In 18/24 patients (75%), only central lymph node metastases were observed, including 8 patients with multifocality (7- pT1bmN1a, 1 -pT3mN1a). Unifocal cancer was detected in 10 cases (7 pT1bN1a, 3 pT3N1a).

Metastases to the lateral cervical lymph node were reported in 6/24 (25%) subjects (3 patients had metastases only to the lateral compartment, and 3 patients also presented with metastases to the central lymph nodes). In this group, multifocality was not observed (**Table 5**).

In the PGcT1bN0M0 group, 2 relapses (1.9%) were observed. Detailed characteristics are presented in **Table 4**. Distant metastasis-free survival was found in all patients.

Early paresis of the recurrent laryngeal nerve assessed on day 1 or day 2 postoperatively was observed in 3 patients (2.9%).

Postoperative hypoparathyroidism (PTH <3 pg/ml) was found in 22 patients (21.2%).

After a month, further supplementation of calcium and vitamin D was maintained in 17 patients (16.3%). Five patients were on continuous supplementation (4.9%).

The comparison of postoperative advancement in PGcT1aN0M0 and PGcT1bN0M0 groups is shown in **Table 5**. Multifocality was more frequently observed in patients in the PGcT1bN0M0 group (p=0.036). Similarly, lymph node metastases were more frequently observed in this group.

Even if we considered only a subgroup of 53 PGcT1aN0M0 patients in whom central lymph nodes were evaluated postoperatively, a significant difference between the groups was found in the prevalence of pN1 (p=0.033). Assuming that in the whole group of 139 patients (PGcT1aN0M0) clinically significant node metastases occurred only in these 5 cases, the statistical difference was even more pronounced (p=0.000) (**Table 5**).

### Stage 4

#### Analysis of Early Postoperative Complications and the Risk of Relapses in Prospective and Retrospective Groups

The RG consisted of 103 patients staged cT1aN0M0 (RG cT1aN0M0) and 91 patients staged cT1bN0M0 (RG cT1bN0M0) were included in the analysis.

In the RGcT1aN0M0 group, 99 patients (96.11%) received adjuvant therapy with radioactive iodine. All patients from the RGT1bN0M0 group were treated with 131 radioactive iodine.

The mean follow-up in the RGcT1aN0M0 group was 12.78 (median 13.88 years), (2.14-16.84 years) and in the RGcT1bN0M0 was 12.59 (median 14.30 years) (3months-16.83 years), respectively.

##### Comparison of the Incidence of Early Postoperative Complications in PG and RG Groups

No significant differences were observed in the incidence of early paresis of the recurrent laryngeal nerves despite the extent of surgery (**Table 6**). This risk of early hypoparathyroidism was over 20% regardless of the fact that in the prospective PGcT1bN0M0 group the extent of prophylactic CND was limited to the unilateral procedure. However, as predicted, LT completely eliminated the risk of postoperative hypoparathyroidism.

**Table 6:** Comparison of the incidence of complications between patients undergoing TT with CND (RG group) and patients treated in the prospective study (PG).

Early complications	RGcT1aN0M0 n=103	PG cT1aN0M0 n=139	RGcT1bN0M0 n=91	PG cT1bN0M0 n=102
Unilateral vocal cord paresis	3 (2.9%)	2 (1.4%)	7 (7.6%)	3 (2.9%)
p	p=0.43		p= 0.129	
Postoperative hypoparathyroidism	22 (21.4%)	0 (0%)	25 (27.5%)	22 (21.2%)
p	p=0.000		p=0.30	

The analysis of complications in the PGcT1aN0M0 group was conducted after the first surgical procedure (LT).

##### Comparison of the Incidence of Relapses in Both PG and RG Groups

In the RG group, the relapse rate was 2 (1.9%) for RGcT1aN0M0, and 5 (5.4%) for RGcT1bN0M0, whereas in the PG group it was 1 (0.7%) for PGcT1aN0M0 and 2 (2%) for PGcT1bN0M0.

No differences were observed in the incidence of relapses between groups regardless of the extent of surgery. However, the mean follow-up in the RG group was significantly longer (p=0.000), and PGT1aN0M0 and RGT1aN0M0 were different in terms of age (p=0.002) (**Table 7**).

**Table 7:** Comparison of the incidence of relapses and the mean time of the follow-up and distribution of sex and age in prospective and retrospective groups.

	RG cT1aN0M0 n= 103	PGcT1aN0M0 n=139	RG cT1bN0M0 n=91	PG cT1bN0M0 n=102
Relapse	2 (1.9%)	1 (0.7%)	5 (5.4%)	2 (2%)
P	ns. p=0.395		ns. p=0.19	
Mean time of the follow-up	12.8 years	6.72 years	12.6 years	6.68 years
P	p=0.000		p=0.000	
Sex Female: Male	92:11	127:12	81:10	92:10
	ns. p=0.755		ns. p=0.787	
Age Mean/Median	45.25/43.47	39.59/37.22	46.05/46.00	45.23/42.67
p	p=0.002		ns. p=0.643	

**Figure 2:**
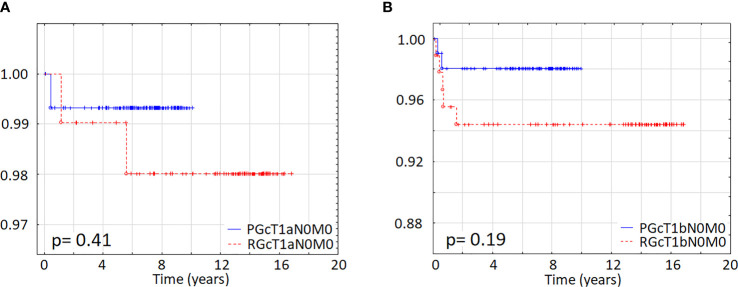
Disease-free survival was not different between the groups **(A)** Comparison between the prospective group (PGcT1aN0M0) and the retrospective population (RGcT1aN0M0) **(B)** Comparison between the prospective group (PGcT1bN0M0) and the retrospective group (RGcT1bN0M0).

Five-year DFS was not statistically different between the prospective and retrospective groups. In the PGcT1aN0M0 group, it was 99.3%, whereas in the RGcT1aN0M0 group it was 99.0% (p =0.41), respectively (**Figure 2A**). In the PGcT1bN0M0 group, it was 98%, while in the RGcT1bN0M0 group it was 94.4% (p = 0.19) (**Figure 2B**).

Of note, apart from 1 relapse in the RGcT1aN0M0 group, which occurred after a 5.6-year follow-up, all other relapses in PG and RG groups were diagnosed during the first 2 years of the follow-up (**Figure 2**). None of the patients died due to thyroid cancer.

## Discussion

The optimal range of surgical treatment of patients with PTC has been the subject of debate between the supporters of TT with prophylactic CND and the supporters of less extensive surgery (5, 20–23) for many years. Currently, the number of the supporters of the second option is growing. For them, one of the most important arguments is the reduction in the risk of postoperative complications with no increase in the risk of failure (2, 24–30). There is no common consensus as to what stage of the disease allows low extensive surgery. The results of our previous retrospective analysis showed that the extent of surgical treatment is important in patients with PTC > 1 cm in diameter (31, 32), which was also observed by other authors (5, 33–35).

Before 2015, Polish guidelines recommended TT with CND when cancer diagnosis was established preoperatively (FNA biopsy). Abandonment of reoperation was possible only if the diagnosis of microcarcinoma was established after thyroid surgery performed due to other indications (8).

We conducted this prospective non-randomized study due to very good prognosis, no worsening of the outcome in the case of the withdrawal from secondary radical thyroidectomy following the diagnosis of microcarcinoma after nodular goiter surgery (8, 25, 26, 31) and the change in ATA recommendations in 2009 (7). Additionally, it was also conducted due to the concern of some Polish medical (and particularly endocrine) societies that expressed the negative attitude to less extensive surgery of PTC.

Participation in the study was voluntary and the subjects had the possibility to withdraw from the trial before surgery. Only 12 cT1aN0M0 patients (7.6%) decided not to undergo LT. Therefore, over 90% of patients accepted less extensive surgery despite being informed about possible negative consequences of this procedure (i.e., possibility of reoperation) in the case of a change in the cancer stage in the postoperative histological examination.

Analyzing all groups of patients i.e., those who requested treatment in accordance with recommendations at that time, those who were recommended LT and patients from the cT1bN0M0 group, it should be noted that preoperative (clinical) disease advancement was confirmed postoperatively in approximately 70% of cases (66.7%, 77.0% and 72.1%, respectively).

The change in the cancer stage was of greatest importance to patients undergoing LT. In 24 of these patients, reoperation was performed due to the results of the postoperative histological examination. The presence of a malignant tumor was observed postoperatively in 8 cases (33%). It seems, however, that a two-stage operation did not negatively affect the prognosis - relapses were not observed in the further follow-up in this group. This procedure is similar to the recommendations in the case of loss of signal in patients undergoing surgery with intraoperative neuromonitoring (36, 37). Postponement of surgical treatment had no influence on thyroid cancer prognosis, which is consistent with other authors (38–40).

It should be noted that due to the change in the TNM classification in 2017 (19, 41), currently the number of patients referred for reoperation would be smaller due to the change in the diagnosis of pT3. Therefore, it is not surprising that a relapse was not observed in the follow-up in patients with this stage of the disease who did not accept reoperation based on the previous TNM classification.

In addition, a detailed discussion with patients concerning the advantages and disadvantages of less extensive surgery was the reason why subjects did not feel disappointed if microcarcinoma was not confirmed in the postoperative examination and another surgical intervention was recommended. Currently, the significance of a joint decision of the patient and the therapeutic team in the diagnostic and therapeutic process is emphasized (2, 28, 42, 43), which is also confirmed by our prospective study.

The comparison of the analysis of the risk of relapse between the PG group and the RG group indicates that the change in the therapeutic strategy did not affect the worsening of the prognosis in patients with low-risk PTC, which is consistent with the observations of other authors (27, 28, 30, 40, 42).

The trial results confirm that metastases to the cervical lymph nodes are the main source of local failure in PTC, which was reported in other studies (2, 44, 45).

Therefore, the experience of the surgeon and the intraoperative assessment of nodal status are crucial. In four cases, it became the reason for widening the extent of surgery during the first surgical intervention. Of note, in our study, most recurrences were related to lateral cervical lymph node metastases.

Therefore, the risk of recurrence resulting from natural tumor biology should be considered regardless of the extent of thyroid resection (46).

Therefore, currently, more attention is paid to the possibility of selecting risk factors which allow for preoperative prediction of the risk of the metastases to the central lymph node compartment (47–52) or the lateral cervical compartment (52–55).

One of these risk factors is related to the tumor diameter. Some studies (48) showed that the risk of lymph node metastasis increased with the tumor diameter of > 2cm. However, our study showed this relationship when the diameter was > 1cm, which is in line with some reports (50, 52) and the results of a meta-analysis (50) which included 31 studies and 37,355 patients with cN0 PTC from seven countries.

Special attention should be paid to the increase in the preoperative detection of lymph node metastases. To achieve it, several studies have recently proposed some preoperative algorithms of management (55–57).

The significance of micrometastases to the lymph nodes remains an open question.

The following question arises: should patients with micrometastases diagnosed after LT be reoperated on? In our study, one such patient was only under follow-up with no recurrence. It seems that in the future the algorithms for management should be established for micrometastases diagnosed in PTC, which is reported in the cases of breast cancer or melanoma.

An important indication for limiting the extent of surgical intervention in low-stage thyroid cancer is to reduce the risk of complications and adverse events (3, 58). Currently, the risk (especially of postoperative hypoparathyroidism) is higher (59, 60) than that reported in the past (61). As early as in 1999, Mitov et al. observed that the rates of complications reported by individual centers were underestimated due to methodological errors and the desire to improve the results (62).

Improvement in the patients’ quality of life was also one of the main reasons for conducting our prospective study. As expected, LT completely eliminated postoperative hypoparathyroidism. This risk, requiring calcium and vitamin D supplementation in patients undergoing TT, was over 20% regardless of the fact that in the prospective PGcT1bN0M0 group the extent of prophylactic central lymphadenectomy was limited to a one-sided operation. This corresponds to the reports of Italian authors (59) who observed that only total abandonment of the central lymphatic dissection reduced the risk of early postoperative hypoparathyroidism. Any extracapsular surgical intervention or LT/thyroidectomy carried a small risk of impaired mobility of the vocal folds (59, 63).

The gradual presentation of the results of the prospective study at scientific conferences (64), and especially at the Thyroid Cancer Conference in Wisla in 2015 (65) was an important voice in the discussion and contributed to the change in the recommendations of surgical treatment of patients with papillary microcarcinoma in Poland. Currently, LT without adjuvant therapy with radioactive iodine is a standard therapeutic option in cT1aN0M0 (43).

It is important to be aware of the differences in the approach to the diagnosis, treatment, course, and outcome of patients with thyroid cancer in different countries (66).

In Europe (and also in Poland), and in Latin America, the indications for TT and adjuvant treatment with radioactive iodine are wider (67–71) than in the United States where the ATA recommendations (2015) allow LT also in patients staged cT2N0M0 (2).

A higher percentage of nodular goiter outside the United States should be also considered at the time of patient qualification for thyroid surgery (72). In Poland, focal lesions in both thyroid lobes are more often reported than in the USA. These findings may be an indication for TT even in the microcarcinoma group and were the reason for collecting a group of only 264 patients with unifocal thyroid cancer of up to 2 cm in diameter who were recommended LT or TT + unilateral CND in our prospective study.

In the recent years, promising results of long-term prospective Japanese studies were reported, which suggested only AS of patients with microcarcinoma (39) and surgical intervention if it is indicated. These results may change the therapeutic approach to patients with cT1aN0M0 disease. Specific indications are currently being developed on patient selection for AS (9–11, 39, 73). The results of the first Italian study (10) confirmed the possibility of conducting AS in Europe. Currently, in our center, we are planning to start a prospective clinical trial related to this issue. Additionally, studies on the possibility of laser therapy and other ablative techniques are reported in patients with thyroid microcarcinoma. It should also be reflected in other recommendations as another therapeutic option along with surgery and AS (13–16).

Our prospective study was a voice in the discussion on the optimization of therapeutic management strategies in patients with low advanced PTC and it had an impact on the change of recommendations in our country (43). The advantage of the study is its prospective nature and a long-term follow-up. However, it has also many limitations (single center and non-randomized). Additionally, it was conducted on relatively small patient groups. It was a response to the change of US recommendations in 2009 (7) and today it possibly could be designed differently, especially for patients staged cT1bN0M0. It seems that LT can also be considered in this group of patients, although it seems that a higher risk of lymph node metastases in this group of patients should be also taken into account.

Furthermore, the clinical and prognostic significance of multifocality is still a controversial issue (74–78). What should be the approach to the diagnosis of multiple small tumor lesions after LT? Should patients be recommended another surgical intervention and iodine therapy?

The following question remains open: will the current change in the TNM classification significantly affect the change in the prognosis in this cancer? (19, 41).

It is also obvious that it is not the tumor diameter itself but also the tumor malignant potential that should impact on further treatment. The development of molecular research, the selection of new prognostic and predictive factors and their analysis in the context of the current classic prognostic factors may facilitate the necessary individualization of patient treatment and decide on patient qualification for more or less aggressive therapies in the future (1, 2, 79, 80). These issues constitute research challenges for the future.

Undoubtedly, the “epidemic” of low-risk PTC results in the de-escalation of diagnosis and treatment. However, caution is exercised not to worsen the excellent prognosis. At the same time, the implementation of new recommendations alone is not sufficient to commonly accept less extensive surgery, which is in line with the most recent American survey. The main aim of this survey was to establish surgical preferences at the time of qualification for LT (node negative low-risk PTC 1-4 cm). The results showed that despite the extension of the 2015 ATA recommendations for LT in cancers ≤ 4 cm in diameter, most surgeons still performed LT, mostly in microcarcinoma and preferred TT in larger tumors, especially in the case of those > 2 cm in diameter (81).

Again, there are also opinions related to the necessity to create a new preoperative risk classification for patients qualified for less extensive surgery. These experts still consider TT to be the treatment of choice for low risk PTC (1-4 cm) (82).

Therefore, it seems that the discussion on safe de-escalation of treatment in low-risk PTC is still open and hence further multicenter studies are warranted.

## Conclusions

The assessment of the results of the prospective clinical trial confirms that less extensive surgery in properly selected patients with low-advanced PTC is both safe and sufficient. LT without adjuvant radioactive iodine therapy is an optimal therapeutic option for patients staged cT1aN0M0. The study has contributed to the introduction of LT as a routine management into clinical practice since 2016 (after the revision of Polish recommendations).

Further studies are warranted to extend the indications for the de-escalation of PTC treatment, including AS or image-guide thermal ablation techniques. Such study results will be useful for the creation of the most recent recommendations.

## Publisher’s Note

All claims expressed in this article are solely those of the authors and do not necessarily represent those of their affiliated organizations, or those of the publisher, the editors and the reviewers. Any product that may be evaluated in this article, or claim that may be made by its manufacturer, is not guaranteed or endorsed by the publisher.

## Data Availability Statement

The datasets presented in this article are not readily available because data is anonymized and according to our rules, available only for patients and treating physician. Request to access the database should be directed to Agnieszka.Czarniecka@io.gliwice.pl.

## Ethics Statement

The studies involving human participants were reviewed and approved by the Bioethics Committee of the Center of Oncology – The Maria Sklodowska-Curie Memorial Institute, Gliwice Branch (no KB/498-18/11). The patients/participants provided their written informed consent to participate in this study.

## Author Contributions 

AC: Conception and study design, data collection, data analysis, literature analysis, and manuscript writing. MZ: Data collection and analysis, statistical analysis, and graphical presentation. GW: Data collection. AM: Data collection and analysis. ES: Data analysis. EC: Data analysis. MO-W: Data and literature analysis. JK: Data collection and analysis. DH-J: Data collections and analysis. BJ: Critical review. All authors contributed to the article and approved the submitted version.

## Conflict of Interest

The authors declare that the research was conducted in the absence of any commercial or financial relationships that could be construed as a potential conflict of interest.

## Publisher’s Note

All claims expressed in this article are solely those of the authors and do not necessarily represent those of their affiliated organizations, or those of the publisher, the editors and the reviewers. Any product that may be evaluated in this article, or claim that may be made by its manufacturer, is not guaranteed or endorsed by the publisher.
